# Anti-Inflammatory and Antioxidant Properties of Black Mulberry (*Morus nigra* L.) in a Model of LPS-Induced Sepsis

**DOI:** 10.1155/2018/5048031

**Published:** 2018-11-07

**Authors:** Karine de Pádua Lúcio, Ana Carolina Silveira Rabelo, Carolina Morais Araújo, Geraldo Célio Brandão, Gustavo Henrique Bianco de Souza, Regislainy Gomes da Silva, Débora Maria Soares de Souza, André Talvani, Frank Silva Bezerra, Allan Jefferson Cruz Calsavara, Daniela Caldeira Costa

**Affiliations:** ^1^Laboratório de Bioquímica Metabólica (LBM), Departamento de Ciências Biológicas (DECBI), Programa de Pós-Graduação em Ciências Biológicas, Universidade Federal de Ouro Preto, Minas Gerais, Brazil; ^2^Laboratório de Farmacognosia, Escola de Farmácia, Programa de Pós-graduação em Ciências Farmacêuticas, Universidade Federal de Ouro Preto, Ouro Preto, Minas Gerais, Brazil; ^3^Laboratório de Imunobiologia da Inflamação, Departamento de Ciências Biológicas (DECBI), Programa de Pós-Graduação em Ciências Biológicas, Programa de Pós-Graduação em Saúde e Nutrição, Universidade Federal de Ouro Preto (UFOP), Ouro Preto, Minas Gerais, Brazil; ^4^Laboratório de Fisiopatologia Experimental (LAFEx), Departamento de Ciências Biológicas (DECBI), Programa de Pós-Graduação em Ciências Biológicas, Universidade Federal de Ouro Preto (UFOP), Ouro Preto, Minas Gerais, Brazil; ^5^Escola de Medicina, Universidade Federal de Ouro Preto (UFOP), Ouro Preto, Minas Gerais, Brazil

## Abstract

Sepsis is a complex disease and is the cause of many deaths worldwide. Sepsis pathogenesis involves a dysregulated inflammatory response with consequent production of inflammatory mediators and reactive species. The production and excessive release of these substances into the systemic circulation trigger various cellular and metabolic alterations that are observed during the disease evolution. Thus, more studies have been carried out to investigate the therapeutic potential of plants such as *Morus nigra* L., popularly known as black mulberry. Studies have shown that plants belonging to the *Morus* genus are rich in secondary metabolites such as flavonoids which are associated with important biological activities as antioxidant and anti-inflammatory actions. Based on this context, the objective of our study was to evaluate the anti-inflammatory and antioxidant properties of *Morus nigra* L. in a sepsis model induced by LPS. Male C57BL/6 mice were distributed in four groups: control, sepsis, sepsis treated with leaf extract of mulberry, and sepsis treated with mulberry pulp. The animals were treated with 100 *μ*L of their respective treatments for twenty-one days. Sepsis was induced at the 21st day with lipopolysaccharide (LPS) by intraperitoneal injection. The animals were euthanized 24 hours after receiving the LPS injection. The data obtained were analyzed in GraphPad Prism 6.0 software. Our results showed that treatment with either extract significantly decreased the number of leukocytes in the bronchoalveolar lavage fluid and serum levels of TNF in septic animals. Regarding the redox status, the treatments significantly decreased the antioxidant activity of the enzyme glutathione peroxidase. Regarding metalloproteinase type 2, it was observed that the treatment with black mulberry pulp was able to significantly reduce the activity of this enzyme concerning the sepsis group. Finally, these results together promoted an increase in the animal's survival that received the black mulberry leaf or pulp extract.

## 1. Introduction

Sepsis is an organ dysfunction that results from a dysregulated host response to infection [1]. The liver plays an important role in sepsis, being essential for microorganism and toxin clearance (such as endoxins) [2]. Endotoxin, as lipopolysaccharide (LPS), a component of the outer membrane of Gram-negative bacteria, interacts with specific receptors on the host effector cells and induces the synthesis of a large number of proinflammatory cytokines. The overproduction of these cytokines can lead to an unregulated inflammatory reaction [3]. However, liver defense responses represent a double-edged sword which can contribute to the destruction and elimination of microbial products and can also lead to liver injury [2].

It is known that there is an interrelation between redox processes and inflammation and that reactive species can activate inflammatory signaling pathways and that inflammatory cells can produce more reactive species, resulting in a vicious cycle leading to a redox and inflammatory disequilibrium [4], and thus may be a determining factor in the sepsis outcome. Thus, the interaction between redox processes and inflammation would be a determining factor for the antioxidant selection with therapeutic potential to minimize systemic damage and improve the septic animal survival. Antioxidant therapies are widely used in diseases that have as main pathogenesis the redox imbalance and inflammatory processes. In this regard, many medicinal plants have an ability to protect the liver due to their antioxidant and anti-inflammatory effects [5].

*Morus nigra* L. (Moraceae family), known as black mulberry, is a tree distributed worldwide [6], including in different regions of Brazil and stands out for its medicinal properties [7]. Many studies have found that black mulberry is rich in polyphenols, flavonoids, and anthocyanins, which are responsible for their antioxidant [8, 9] and anti-inflammatory activities [10, 11]. Based on the fact that the fruits and leaves of *Morus nigra* have anti-inflammatory and antioxidant potential as well as on the fact that due to being a good therapeutic candidate to minimize systemic complications due to sepsis, it should contain both anti-inflammatory and antioxidant characteristics, the objective herein was to verify the potential of the leaf and pulp of *Morus nigra* in preventing or minimizing redox and inflammatory imbalance induced by sepsis.

## 2. Materials and Methods

### 2.1. Botanical Material

The aerial parts of *Morus nigra* were collected in the city of Ouro Preto, Minas Gerais, Brazil, in the year 2012. The specimen was identified by the number OUPR 27087 and deposited in the herbarium José Badini of the Federal University of Ouro Preto (UFOP).

### 2.2. Preparation of the Pulp and Hydroethanolic Leaf Extracts of *Morus nigra*

The ripe fruits were collected in September 2015, and the black mulberry extract was obtained after the fruits were pressed and subjected to filtration. The filtrate was stored in a freezer at −80°C. The black mulberry leaves were dehydrated in a drying oven at 37°C and then crushed. The extraction was performed with a hydroalcoholic solution in a ratio of 1 : 1 by percolation for 24 hours at room temperature. The filtrate was concentrated in a rotary evaporator. The dried residue was dissolved in filtered water to the final concentration of 150 mg∙mL^−1^.

### 2.3. RP-UPLC-DAD-ESI-MS Analyses

Chemical characterization of the fluid extract of fruits and the hydroalcoholic extract of *Morus nigra* leaves was performed by ultra performance liquid chromatography (UPLC) coupled to a diode arrangement detector and mass spectrometry. The hydroethanolic extract was resuspended in a 98 : 2 (methanol : water) solution and then filtered through a 0.20 *μ*m PVDF syringe filter. Subsequently, it was injected into the liquid chromatograph. Chromatographic separation was performed in ACQUITY UPLC BEH RP-18 (1.7 *μ*m, 50 × 2 mm i.d.) (Waters). The mobile phase consisted of water 0.1% formic acid (solvent A) and acetonitrile 0.1% formic acid (solvent B). The elution protocol was 0–11 min and linear gradient was ranging from 5% to 95% B. The flow rate was 0.3 mL∙min^−1^, and the sample injection volume was 4.0 *μ*L. Analyses were performed using an ACQUITY UPLC (Waters) ion trap mass spectrometer in the following conditions: positive and negative ion mode; capillary voltage, 3500 V; capillary temperature, 320°C; source voltage, 5 kV; vaporizer temperature, 320°C; corona needle current, 5 mA; and sheath gas, nitrogen, 27 psi. Analyses were run in the full scan mode (100–2000 Da). The ESI-MS/MS analyses were additionally performed in an ACQUITY UPLC (Waters) with argon as the collision gas, and the collision energy was set at 30 eV. The UV spectra were registered from 190 to 450 nm.

### 2.4. Animal Protocol

Male C57BL6 mice were separated into 4 experimental groups: control group (C), sepsis group (S), sepsis group treated with leaf extract of *Morus nigra* (SL), and sepsis group treated with pulp of *Morus nigra* (SP). During 21 days, groups C and S received 100 *μ*L of water per gavage and the SL and SP groups received 100 *μ*L at a dose of 500 mg∙kg^−1^ of leaf extract and pulp extract, respectively, of *Morus nigra* [12, 13]. At the last day of the test, the animals received the treatments and 1 hour after the controls received intraperitoneal injection with saline solution and the other groups received LPS injection at a dose of 10 mg∙kg^−1^ (0111: B4, Sigma). The animals were euthanized 24 hours after sepsis induction, and through the trachea cannulation, the animals' lungs were perfused with saline solution (0.9% NaCl) to collect bronchoalveolar lavage. Blood and liver samples were collected and stored in the freezer at −80°C for further analysis, and the mortality rate was measured.

### 2.5. Biochemical Parameters

Serum glucose was obtained from blood samples taken from the animals' tail vein moments before their euthanasia using the Accu-Check® glycosimeter. Platelet quantification was obtained from a hemogram performed in an automated equipment.

### 2.6. Expression of Inflammatory Mediators

The real-time quantitative RT-PCR assay (RT-PCR) technique was used to evaluate the cytokine expression in liver samples. RNA extraction was performed using the SV Total RNA Isolation System Kit (Promega Corporation, catalog # Z3100). The concentration and purity of RNA in the samples subjected to extraction were evaluated by tests in NanoVue (GE Healthcare, UK) using the A260/280 ratio. The cDNA molecule was formed using High-Capacity cDNA Reverse Transcription Kit (Applied Biosystems, catalog # 4368814) and oligo (dT) which is capable of converting 1 *μ*g of RNA to cDNA. Primers for IL-1*β*, IL-6, and IL-10 were constructed from the Primer-BLAST program. DNA amplification, detection, and quantification reactions occurred at 50°C for 2 min, 95°C for 10 min in 40 cycles at 95°C for 15 sec, and 60°C for 1 min using the ABI Prism 7300 Sequence Detector (Applied Biosystems, CA, USA). Data were analyzed by comparative *C*_*T*_ method. Target gene expression was determined relative to the expression of *β*-actin gene. All analyses were performed in triplicate.

### 2.7. Analysis of Cytokine Level

The serum cytokine concentrations and also in hepatic tissue were determined using enzyme-linked immunosorbent assay kits ((PeproTech), Murine IL-6 Standard ABTS ELISA Development Kit (catalog # 900-K50), Murine TNF-*α* Standard ABTS ELISA Development Kit (catalog # 900-K54), Murine IL-1*β* Mini ABTS ELISA Development Kit (catalog # 900-M47), and Murine IL-6 Mini ABTS ELISA Development Kit (catalog #900-M50)), according to the manufacturer's instructions.

### 2.8. Analyses of Antioxidant Defenses

#### 2.8.1. Catalase Activity

The hepatic tissue (100 mg) was fractionated and homogenized with 1000 *μ*L of phosphate buffer (0.1 M, pH 7.2) and subsequently centrifuged at 10.000 rpm for 10 minutes. For the assay, 10 *μ*L of the obtained supernatant and 990 *μ*L of peroxide solution (6%) were mixed. Reading was performed in a spectrophotometer at 240 nm, and enzyme activity was measured by decaying the absorbances every 30 seconds during 3 minutes of reading as described previously by Aebi [14]. Hydrolysis of 1 *μ*mol of H_2_O_2_ per min was equivalent to one unit (U) of catalase.

#### 2.8.2. Superoxide Dismutase Activity

The superoxide dismutase enzyme (SOD) activity was measured indirectly. As described previously by Marklund and Marklund [15], pyrogallol undergoes autoxidation producing the superoxide anion (O^•−^). The SOD enzyme is able to compete for the superoxide anion by decreasing the MTT reduction ([3-(4,5-dimethyl-2-thiazolyl)-2,5-diphenyl-2H-tetrazolium bromide]) and consequently the formazan crystal formation. The reading was performed in a spectrophotometer at 570 nm. The results were expressed as U SOD/mg of protein, where one unit of SOD is defined as the amount of enzyme required for 50% inhibition of MTT reduction.

#### 2.8.3. Total Glutathione, Oxidized Glutathione, and Reduced Glutathione

Total glutathione content (GSHt) was determined by a kinetic assay using a protocol adapted from the commercial sigma kit (Sigma, catalog # CS0260). In this assay, DTNB [5,5′-dithiobis (2-nitrobenzoic acid)] is reduced to TNB (2-nitro-5-thiobenzoate) and this reduction is directly proportional to the tripeptide concentration in the assessed tissue since reduced glutathione (GSH) is the cofactor of this reaction. To determine oxidized glutathione (GSSG) concentration, homogenate derivatization with 2,2′,2′′-nitrilotriethanol, tris(2-hydroxyethyl)amine (TEA), and vinylpyridine was performed. The concentrations of GSHt and GSSH were obtained by a standard curve performed for each of these assays. The GSH concentration was obtained by subtracting the oxidized glutathione value from the total glutathione concentration.

#### 2.8.4. Glutathione Reductase Activity

The activity of the glutathione reductase enzyme was measured by the Glutathione Reductase Assay Kit (Abcam, catalog # ab83461). The assay is based on the glutathione reduction by NADPH in the presence of glutathione reductase. The reading is carried out at 412 nm.

#### 2.8.5. Glutathione Peroxidase Activity

The activity of the enzyme glutathione peroxidase was determined using the Glutathione Peroxidase Activity Kit (Assay Designs Inc., catalog # 900-158). The assay is based on the oxidation of GSH to GSSG by the enzymatic action of glutathione peroxidase coupled to the recycling of GSSG to GSH by glutathione reductase. The absorbance decay caused by the oxidation of the reduced coenzyme NADPH to NADP is measured at 340 nm being indicative of the activity of glutathione peroxidase since this enzyme is a limiting step of this reaction.

### 2.9. Gelatin Zymography

The activity of types 2 and 9 metalloproteinases was evaluated by electrophoresis in 8% polyacrylamide gel containing 2 mg∙mL^−1^ of porcine gelatin (Sigma Chem. Co., St. Louis, USA). Samples of hepatic extract at the concentration of 30 *μ*g of protein were applied to the gels, and the electrophoresis was performed for 120 minutes at 100 V. After the run, the gels were washed with 10% Triton-X solution and incubated in buffer composed of 50 mM of tris, 150 mM of NaCl, 5 mM of CaCl_2_, and 0.05% of NaN_3_ (pH 7.5) for 36 h at 37°C. After incubation, the gels were stained with 0.05% coomassie brilliant blue G-250 for 3 h and decolorized with methanol and acetic acid solution (4%–8%) as previously described by Sung et al. [16].

### 2.10. Histological Analysis

Fragments of the animal livers were fixed in 4% buffered formalin, and for the preparation of slides, they were subjected to serial dehydration with alcohols of decreasing concentrations and were subsequently immersed in paraffin. The paraffin sections of approximately 4 *μ*m were obtained in microtome, assembled, and stained by the hematoxylin and eosin (H&E) technique, to visualize the tissue structure. The photomicrographs were obtained using a Leica optical microscope coupled to the DM5000 digital camera using a 40x objective lens, and the morphometric analyzes were performed in the Leica Application Suite (Germany) analysis software.

### 2.11. Statistical Analysis

The normality of the distribution of each variable was assessed by means of the Kolmogorov-Smirnov test. Data that presented a normal distribution were analyzed through univariate analysis of variance (ANOVA-one way), followed by Bonferroni's posttest to determine the differences among the C, SL, and SP groups in relation to the S group. Data were expressed as mean ± standard error. Differences were considered significant when *p* < 0.05. All tests were performed on GraphPad Prism 6.0 software for Windows (San Diego, California, USA).

## 3. Results

### 3.1. RP-UPLC-DAD-ESI-MS Analyses

**Figure 1:**
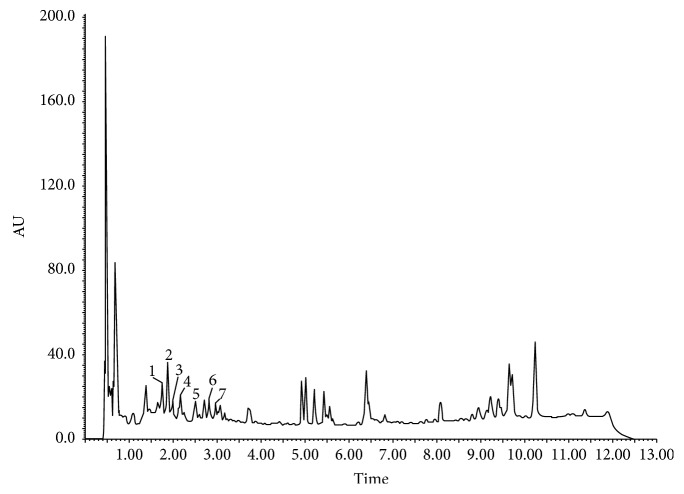
Profile of the leaf extract of *Morus nigra* L. 1: 3-O-Caffeoylquinic acid; 2: 4-O-caffeoylquinic acid; 3: 5-O-caffeoylquinic acid; 4: 6-hydroxy-luteolin-7-O-rutenoside; 5: quercetin-3-O-furanosyl-2-ramosil; 6: quercetin-3-O-rutenoside; 7: quercetin 3-O-glycoside.

**Figure 2:**
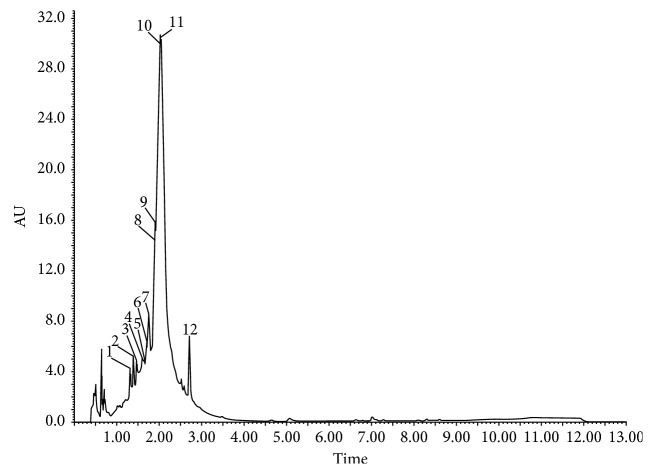
Profile of *Morus nigra* L. pulp. 1: 3-O-Caffeoylquinic acid; 2: 4-O-caffeoylquinic acid; 3: delphinidin 3-O-rutinoside; 4: 5-O-caffeoylquinic acid; 5: delphinidin 7-O-rutinoside; 6: delphinidin-3-O-glucoside; 7: cyanidin 3-O-glucoside; 8: delphinidin 7-O-glycoside; 9: cyanidin-O-glycosyl-aminosulfonate; 10: quercetin 3-O-rutinoside; 11: quercetin 3-O-glycoside/quercetin 7-O-glycoside; 12: quercetin 7-O-glycoside/quercetin 3-O-glycoside.

The results presented in Figures 1 and 2 and Tables 1 and 2 show the phenolic substances which have been identified in pulp extract and leaf extract, respectively, of *Morus nigra* including the glycosides of flavonoids. The mass spectra obtained were compared with the results described in the literature. The searches were performed on the data basis available for the *m*/*z* ratio obtained in the spectra.

### 3.2. Effect of *Morus nigra* on Survival and Biochemistry Parameters in Septic Mice

**Figure 3:**
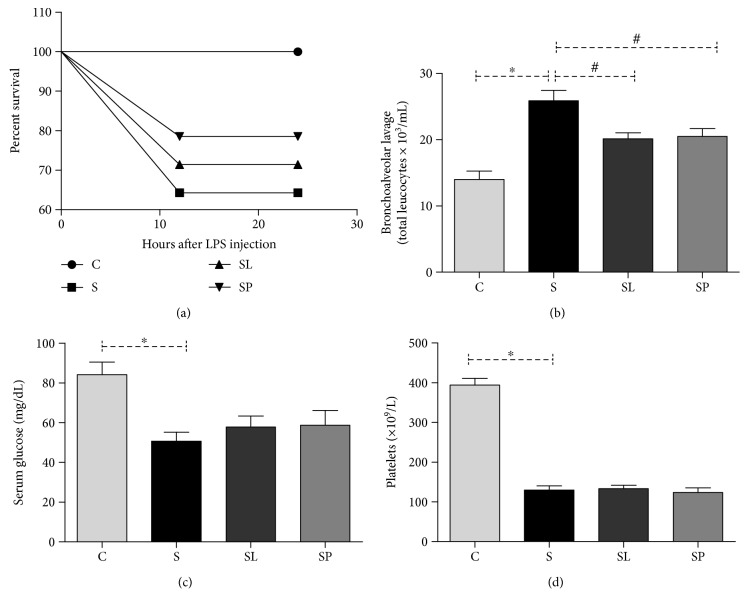
Effect of treatments with a pulp or leaf extract of *Morus nigra* on sepsis-induced percent survival (a) in C57BL/6 mice after 24 hours of LPS injection, influx cellular in bronchoalveolar lavage (b), serum glucose (c), and platelets (d). All data are shown as mean ± SEM. ^∗^*P* < 0.05 indicates a significant difference between group S (sepsis) and group C (control). ^#^*P* < 0.05 indicates a significant difference between S, SL (sepsis + leaf extract of mulberry), and SP (sepsis + mulberry pulp) groups.

It is possible to observe that the animals pretreated with pulp and leaf extracts had a longer survival (78.5% and 71.4%, respectively) than the animals that did not receive treatment (Figure 3(a)). In addition, it was observed that the sepsis animal group showed a decrease in the total number of platelets (Figure 3(d)) and hypoglycemia (Figure 3(c)), in addition to an increase in the number of total leucocytes in the bronchoalveolar lavage fluid (BLF) (Figure 3(b)), being all of these manifestations characteristic of sepsis. Extracts from *Morus nigra* leaves as well as fruit pulp were able to decrease the number of inflammatory cells.

### 3.3. Effect of *Morus nigra* on the Inflammatory Profile in the Sepsis

**Figure 4:**
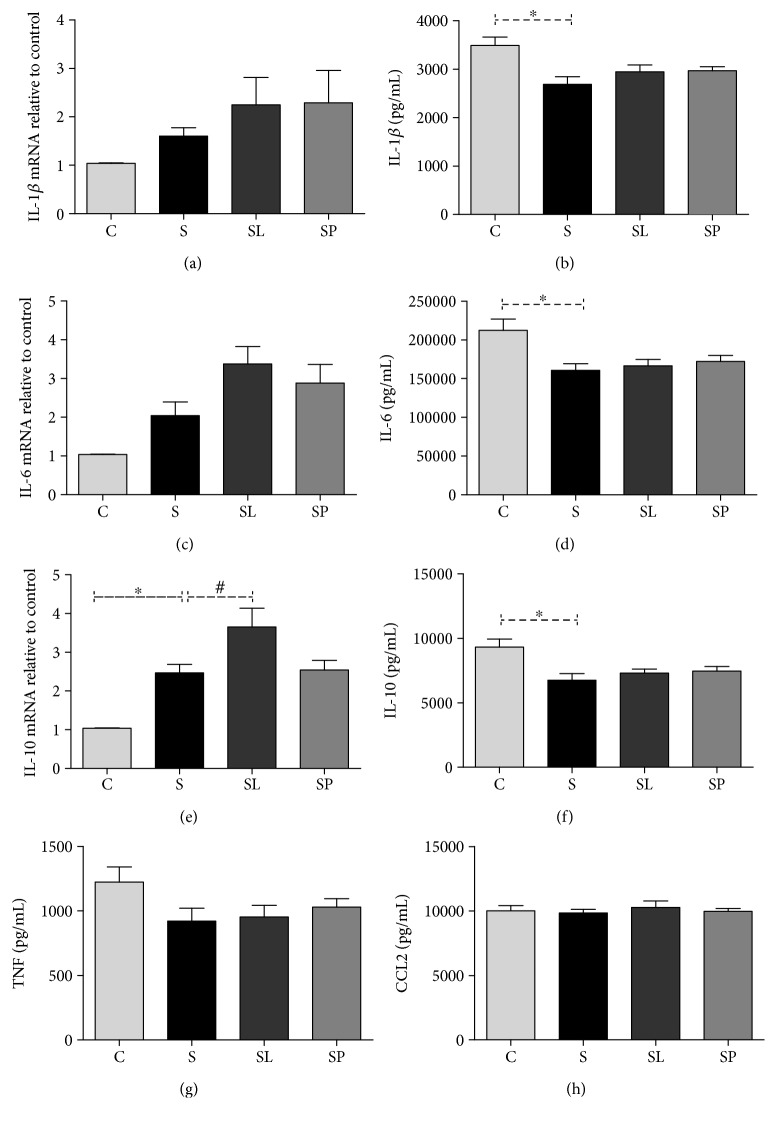
Effects of pulp and leaf extracts on expression and concentration of cytokines and interleukins in the liver samples from the C, S, SL, and SP groups. (a) IL-1*β* expression, (b) IL-1*β* levels, (c) IL-6 expression, (d) IL-6 levels, (e) IL-10 expression, (f) IL-10 levels, (g) TNF levels, and (h) CCL2 levels. All data are shown as mean ± SEM. ^∗^*P* < 0.05 indicates a significant difference between group S (sepsis) and group C (control). ^#^*P* < 0.05 indicates a significant difference between S, SL (sepsis + leaf extract of mulberry), and SP (sepsis + mulberry pulp) groups.

Regarding the regulation of the expression of inflammatory mediators in the liver, no significant changes were observed in gene expression of IL-1*β* (Figure 4(a)) and IL-6 (Figure 4(c)) in any of the experimental groups; however, a significant decrease was observed in hepatic levels of IL-1 *β* (Figure 4(b)) and IL-6 (Figure 4(c)) in septic animals. Concerning IL-10, an increase in the gene expression of this cytokine was observed in the sepsis group compared to the control as well as an increase in the expression of this gene in the group treated with pulp of black mulberry in relation to the sepsis group (Figure 4(e)); however, a decrease was observed in hepatic levels of IL-10 in septic animals compared to the control (Figure 4(f)). No alterations were observed in the levels of TNF and CCL2 in any of the experimental groups (Figures 4(g) and 4(h), respectively).

**Figure 5:**
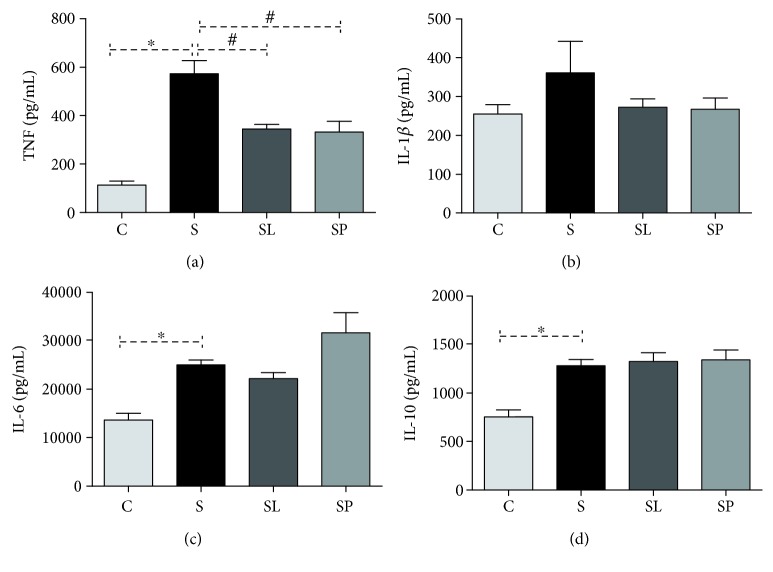
Effects of pulp and leaf extracts on serum cytokine levels. (a) TNF, (b) IL-1*β*, (c) IL-6, and (d) IL-10. All data are shown as mean ± SEM. ^∗^*P* < 0.05 indicates a significant difference between group S (sepsis) and group C (control). ^#^*P* < 0.05 indicates a significant difference between S, SL (sepsis + leaf extract of mulberry), and SP (sepsis + mulberry pulp) groups.

In relation to the systemic profile, a significant increase was observed in the levels of TNF, IL-6, and IL-10 in animals of the sepsis group compared to the control (Figures 5(a), 5(c), and 5(d), respectively). The treatment with the extracts of leaves and pulp reduced the levels of TNF in relation to the septic group without treatment (Figure 5(a)).

No significant alterations were observed in the level of IL-1*β* in any of the experimental groups (Figure 5(b)).

### 3.4. Effect of *Morus nigra* on the Redox Status in the Sepsis Model

**Figure 6:**
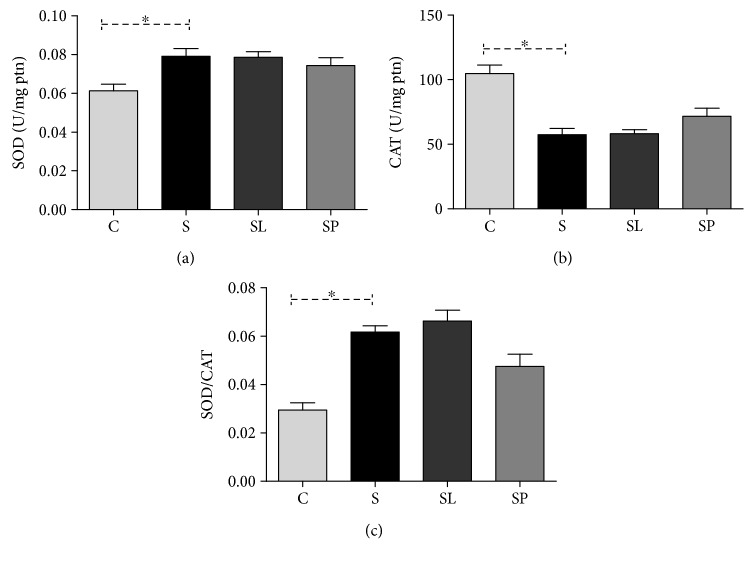
Effects of pulp and leaf extracts on the liver antioxidant enzymes. (a) SOD activity, (b) CAT activity, and (c) SOD/CAT ratio. All data are shown as mean ± SEM. ^∗^*P* < 0.05 indicates a significant difference between group S (sepsis) and group C (control). ^#^*P* < 0.05 indicates a significant difference between S, SL (sepsis + leaf extract of mulberry), and SP (sepsis + mulberry pulp) groups.

The results in this study show that animals with sepsis showed an increase in the superoxide dismutase (SOD) activity (Figure 6(a)) and a decrease in the catalase (CAT) activity (Figure 6(b)), which resulted in an increase in SOD/CAT (Figure 6(c)). No significant change was observed regarding the groups treated with the extract of leaves and pulp of black mulberry.

**Figure 7:**
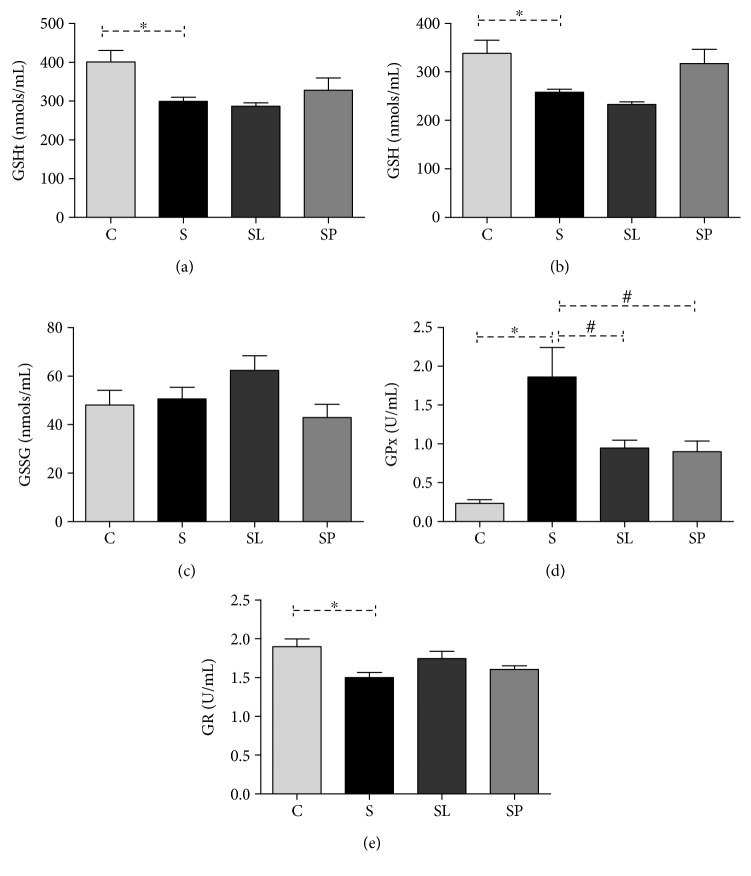
Effects of pulp and leaf extracts on hepatic glutathione metabolism. (a) Total glutathione (GSHt), (b) reduced glutathione (GSH), (c) oxidated glutathione (GSSG), (d) glutathione peroxidase activity (GPx), and (e) glutathione reductase activity (GR). All data are shown as mean ± SEM. ^∗^*P* < 0.05 indicates a significant difference between group S (sepsis) and group C (control). ^#^*P* < 0.05 indicates a significant difference between S, SL (sepsis + leaf extract of mulberry), and SP (sepsis + mulberry pulp) groups.

Concerning the glutathione metabolism, a reduction was observed in the total glutathione content (Figure 7(a)) and in the reduced fraction (Figure 7(b)), in addition to a significant increase in GPx activity (Figure 7(d)) and a decrease in the GR activity (Figure 7(e)) in the sepsis group when compared to the control group. The treatment with the black mulberry pulp was able to restore the GSH levels to levels close to the control. In addition, a reduction was observed in the activity of GPx in the SL and SP groups. No significant differences were also observed among groups C, SL, and SP in relation to the activities of GR and GSSG levels (Figure 7(c)).

### 3.5. Effect of *Morus nigra* on the Liver Damage in the Sepsis Model

**Figure 8:**
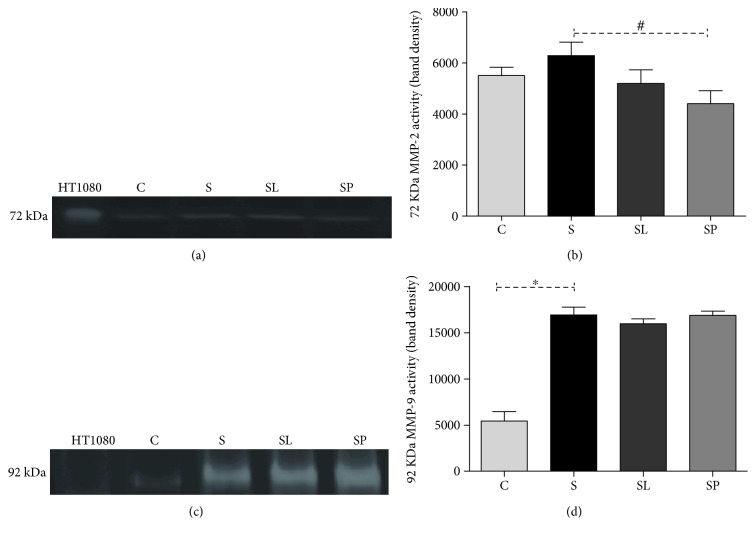
(a) Representative image of MMP-2 bands in the gel. (b) Effects of pulp extract and leaf extract on MMP-2 activity in hepatic homogenate. (c) Representative image of MMP-9 bands in the gel. (d) Effects of pulp and leaf extracts on MMP-9 activity in hepatic homogenate. C, S, SL, and SP; HT-1080 (Std.). All data are shown as mean ± SEM. ^∗^*P* < 0.05 indicates a significant difference between group S (sepsis) and group C (control). ^#^*P* < 0.05 indicates a significant difference between S, SL (sepsis + leaf extract of mulberry), and SP (sepsis + mulberry pulp) groups.

The results of Figure 8 show a decrease in the MMP2 activity in the sepsis animal group treated with pulp of black mulberry in relation to the nontreated sepsis group. In addition, it is possible to observe an increase in the MPP9 activity of septic animals compared to the control and no change was observed in animals treated with *Morus nigra*.

**Figure 9:**
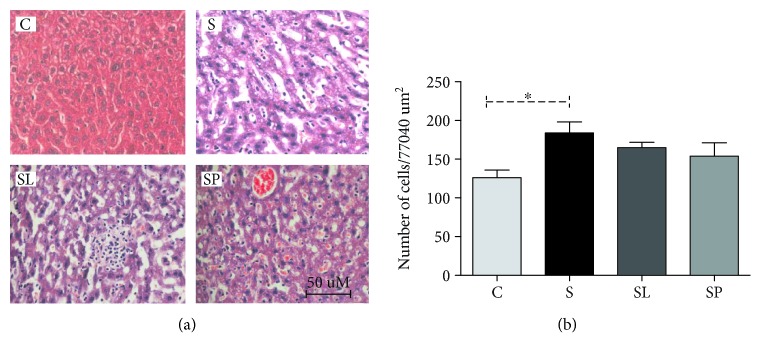
(a) Representative photomicrograph of hepatic tissue showing increased influx of leukocytes in septic mice (H&E × 40). (b) Effect of pulp extract and leaf extract on the number of leukocytes in the hepatic tissue of septic mice. All data are shown as mean ± SEM. ^∗^*P* < 0.05 indicates a significant difference between group S (sepsis) and group C (control). ^#^*P* < 0.05 indicates a significant difference between S, SL (sepsis + leaf extract of mulberry), and SP (sepsis + mulberry pulp) groups.

It was observed that there was no significant change in the liver histoarchitecture tissue in all the experimental groups (Figure 9(a)); however, a significant increase of inflammatory cells in the sepsis animal group and a reduction of 21% of inflammatory cells in the sepsis animal group treated with the pulp were observed, although this difference is not significant (Figure 9(b)).

## 4. Discussion

The present study demonstrated that pretreatment with black mulberry extracts was important to reduce the imbalance in the inflammatory and redox status of animals with sepsis induced by LPS, which could be seen through the reduction in the number of inflammatory cells present in the bronchoalveolar lavage, by the decrease in serum levels of TNF, GPx, and MMP2 activities and in the restoring of the GSH levels. These results analyzed in conjunction may justify the higher survival rate of animals that were pretreated with *Morus nigra*, suggesting that this plant presents a therapeutic potential to minimize the damage caused by sepsis.

It is known that leaves and fruits of *Morus nigra* exhibit a wide spectrum of biological activities, such as antimicrobial, antioxidant, and immunoregulatory properties [17, 18], due to the fact that they contain high levels of phenolic compounds [19]. Previous results from our research group demonstrated that *M. nigra* leaves present a higher content of phenolic compounds and antioxidant activity measured by the ability to neutralize the DPPH radical when compared to its fruits, besides a distinct chromatographic profile between the extract of leaves and that of pulp of black mulberry [12]. In this study, a distinct chromatographic profile was also observed between the two preparations, being that in the fruit samples, the flavonoids delphinidin and cyanidin were observed. Delphinidin is derived from catechin and epicatechin [20], and it is widely found in plant-based food [21]. Its anti-inflammatory activities are well reported [22]. Cyanidin is an anthocyanin that has been extensively studied due to its antioxidant protection through the modulation of the Nrf2 transcription factor [23]. Although the berry production is more common in the Northern Hemisphere, the chromatographic profile observed in this study is in agreement with other studies of berries grown in Brazil [24].

It is known that the anti-inflammatory and antioxidant properties of *Morus nigra* can be a determinant factor in the inflammatory processes and redox modulation induced by sepsis. The inflammatory mediator activation in sepsis results in a metabolic imbalance [25]. Hypoglycemia observed in our study of a group of septic animals has also been observed in other studies [26, 27]. It is known that LPS is responsible to inhibit hepatic gluconeogenesis [28, 29]. In the same way, platelets are recognized to play an important role in innate and adaptive immunity [30], protecting against LPS-induced sepsis [31]. The treatment with *Morus nigra* did not alter the hypoglycemia and thrombocytopenia observed in septic animals. However, another well-studied parameter in sepsis is the inflammatory cell infiltration into the lungs. LPS induces lung microvascular injury, including leukocyte accumulation in the lung tissue, which plays an important role in the sepsis severity [32]. The results of this study show that the treatment with *Morus nigra* was able to reduce the inflammatory infiltrate in the lung. The presence and manifestations of immune activation are not uniform across the body spaces and organs. Thus, it is likely that a focus of infection at one site can produce organ damage at a distant site [33]. Based on this, levels of pro- and anti-inflammatory cytokines in the serum and the liver were evaluated. The liver has an important role in bacterial and toxin clearance in sepsis [34]. Moreover, a major mechanism of sepsis immunosuppression is in liver desensitization, where the production of proinflammatory cytokines involves the concomitant induction of anti-inflammatory mediators, such as IL-10, in an attempt to protect the liver from injury [35]. The results showed an increase in the gene expression of IL-10 and a decrease in hepatic levels of IL-10, IL-6, and IL-1*β* in septic animals when compared to the control, and the treatment with *Morus nigra* did not alter either the gene expression or the hepatic levels of cytokines. Analyzing these results, it is possible to infer that there was no association between the gene expression of cytokines and their hepatic levels, suggesting that posttranscriptional modification may be responsible for determining the levels of cytokines in the liver. Another point to be considered is that the host response to infection is a time- and space-compartmentalized process that involves the inflammatory cytokine release that causes organ damage, being that the balance between proinflammatory and anti-inflammatory responses is a vital process in the liver during sepsis [35].

The events underlying the inflammatory response to lipopolysaccharide include the LPS detection by pattern recognition receptors, followed by the coordinated expression of proinflammatory cytokines (TNF, IL-1*β*, and IL6) [36]. Regarding the serum levels of cytokines, an increase of TNF, IL-1*β*, and IL6 was observed and both treatments were efficient to reduce the serum levels of TNF. TNF-*α* is key sepsis mediator [37] and is increased in circulation following LPS administration [38]; thus, the serum decrease of TNF can have an important role in the sepsis outcome.

The antioxidant profile in the animals' liver was assessed as a means of evaluating the redox imbalance caused by sepsis and the effects of *Morus nigra* on the antioxidant defenses. Analyzing the ratio between the superoxide dismutase and catalase activities, it was observed that it remained significantly increased in animals belonging to the septic groups. This demonstrates that the increase in the SOD activity was not followed by an increase in the CAT activity. In this condition, the SOD activity does not usually protect the cell from a possible redox imbalance; on the contrary, its activation results in an increase in hydrogen peroxide production that can mediate damage to the membranes and other biomolecules [39]. The glutathione system is another important component of the cellular antioxidant defense. Therefore, the enzyme glutathione peroxidase (GPx) has been proved to be an important oxidative stress biomarker in septic patients playing a critical role in the vital organ and tissue protection against oxidative damage. The increase in the SOD enzyme activity in all septic groups and the consequent increase in the hydrogen peroxide production were not followed by an increase in the catalase enzyme which is responsible for catalyzing the hydrolysis of this hydrogen peroxide into water and oxygen molecules. Thus, the accumulated peroxide may have been diverted to the glutathione system by the action of the enzyme glutathione peroxidase. This would justify the increase in the activity of this enzyme in septic animals. Many studies have already shown that LPS can raise the antioxidant enzyme activity as a response to the increase of reactive species caused by this substance [40, 41]. On the other hand, the activity of this enzyme in animals that received the treatments may have been reduced by the direct antioxidant action of phenolic compounds present in the two preparations of *Morus nigra* which were used in this study, in which the polyphenols are capable of reacting with the reactive species neutralizing their oxidant action in the organism [42].

It is known that LPS is able to induce the increase in the activity of several matrix metalloproteinases (MMPs), mainly MMP-2 and MMP-9 [43]. MMPs are a family of calcium- and zinc-dependent endopeptidases responsible for remodeling and degradation of extracellular matrix of basal membrane [44]. Our results show an increase in the MMP9 activity without a change in the MMP2 activity in the animals' liver of the sepsis group. It is known that MMP-2 is a constitutive expression enzyme, slightly responsive to the majority of stimuli, whereas MMP-9 is inducible, being considered a marker of systemic inflammation in animals [45]. Thus, it is possible to infer that the pretreatment with *Morus nigra* is able to inhibit the MMP2 constitutive expression without changing the MMP9 activity.

Concerning the liver histological analysis, a significant increase was observed in the influx of leukocytes in this tissue in septic animals when compared to control animals. It was also observed that the treatments were not capable of changing this parameter. The analysis of the liver tissue architecture revealed an apparent increase of sinusoidal capillaries in septic animals that can be explained by the influx of leukocytes in the organ. Sakurai et al. (2017) [46] also observed dilatation and congestion of the sinusoidal capillaries in septic animals in an experimental model of sepsis induced with LPS in the first hours after induction.

The results of this study made evident the complexity of sepsis, showing that this is a disease capable of compromising the function of several organs, especially the liver. This study also showed that the treatment with the extracts of leaves and the pulp of *Morus nigra* produced beneficial effects on the modulation of important parameters that are normally altered in sepsis. This shows that the chemical compounds present in both preparations can modulate, at least in part, the damage caused by sepsis.

## Figures and Tables

**Table 1:** Substances identified in the pulp of *Morus nigra* by LC-DAD-ESI-MS.

Peak	Compound	RT (min)	UV (nm)	LC-MS [M − H]^−^ (*m/z*)	LC–MS [M + H]^+^ (*m/z*)
1	3-*O*-Caffeoylquinic acid	1.76	323.1	353.3 (191.2; 179.3; 134.8)	355.4
2	4-*O*-Caffeoylquinic acid	1.88	321.1	353.4 (191.3; 179.2; 135.1)	355.7
3	5-*O*-Caffeoylquinic acid	1.99	323.1	353.5 (190.6; 179.2; 135.4)	355.9
4	6-Hydroxy-luteolin-7-*O-*rutenoside	2.17	265.3; 327.8	609.5 (301.1)	611.5
5	Quercetin-3-*O*-furanosyl-2^″^-ramnosyl	2.53	264.1; 357.8	579.2 (433.6; 301.0; 277.3)	581.7
6	Quercetin-3-*O*-rutenoside	2.70	255.3; 353.2	609.2 (301.0; 161.2)	611.2
7	Quercetin 3-*O*-glucoside	2.86	255.2; 355.3	463.1 (301.3)	465.2

TR (min): retention time in minutes; UV (nm): ultra violet in nanometers; LC-MS [M − H]^−^ (*m*/*z*): liquid chromatography coupled to negative-mode mass spectrometry; LC-MS [M + H]^+^ (*m*/*z*): liquid chromatography coupled to mass spectrometry in positive, mass/charge ratio.

**Table 2:** Substances identified in the extract of *Morus nigra* by LC-DAD-ESI-MS.

Peak	Compound	RT (min)	UV (nm)	LC-MS [M − H]^−^ (*m/z*)	LC-MS [M + H]^+^ (*m/z*)
1	3-*O*-Caffeoylquinic acid	1.46	323.1	353.4 (191.1; 179.0; 134.8)	355.39
2	4-*O*-Caffeoylquinic acid	1.74	321.1	353.4 (191.1; 179.0; 135.2)	355.72
3	Delphinidin 3-*O*-rutinoside	1.88	280.4	611.3 (285.0; 302.8; 474.8)	611.3
4	5-*O*-Caffeoylquinic acid	1.93	323.1	353.4 (190.8; 179.0; 135.0)	355.92
5	Delphinidin 7-*O*-rutinoside	1.98	281.1	611.3 (285.0; 302.8; 474.8)	611.3
6	Delphinidin 3-*O*-glucoside	1.97	280.4	465.3 (285.2; 301.3; 329.2)	465.34
7	Cyanidin 3-*O*-glucoside	1.99	280.1	447.3	449.38 (287.0)
8	Delphinidin 7-*O*-glucoside	2.05	281.4	465.3 (285.2; 301.3; 329.3)	465.35
9	Cyanidin 3-*O*-glucosyl-ramnoside	2.10	281.1	593.2	595.42 (449.1; 287.1)
10	Quercetin 3-*O*-rutinoside	2.70	255.4; 354.4	609.8 (301.3; 163.1)	611.60
11	Quercetin 3-*O*-glucoside/Quercetin 7-*O*-glucoside	2.71	255.1; 359.1	463.5 (301.1)	465.35 (303.2)
12	Quercetin 7-*O*-glucoside/Quercetin 3-*O*-glucoside	2.82	254.1; 358.1	463.2 (301.0)	465.48 (303.4)

TR (min): retention time in minutes; UV (nm): ultra violet in nanometers; LC-MS [M − H]^−^ (m/z): liquid chromatography coupled to negative-mode mass spectrometry; LC-MS [M + H]^+^ (m/z): liquid chromatography coupled to mass spectrometry in positive, mass/charge ratio.

## Data Availability

The results are available in the manuscript itself, but if any reader has any doubts, we are willing to respond.
